# Learning-based endovascular navigation through the use of non-rigid registration for collaborative robotic catheterization

**DOI:** 10.1007/s11548-018-1743-5

**Published:** 2018-04-12

**Authors:** Wenqiang Chi, Jindong Liu, Hedyeh Rafii-Tari, Celia Riga, Colin Bicknell, Guang-Zhong Yang

**Affiliations:** 1 Hamlyn Centre for Robotic Surgery, Imperial College London, London, SW7 2AZ UK; 2 Department of Surgery and Cancer, Imperial College London, St Marys Hospital, London, W2 1NY UK

**Keywords:** Robotic catheterization, Robotic surgery, Human–robot collaboration, Imitation learning

## Abstract

**Purpose:**

Endovascular intervention is limited by two-dimensional intraoperative imaging and prolonged procedure times in the presence of complex anatomies. Robotic catheter technology could offer benefits such as reduced radiation exposure to the clinician and improved intravascular navigation. Incorporating three-dimensional preoperative imaging into a semiautonomous robotic catheterization platform has the potential for safer and more precise navigation. This paper discusses a semiautonomous robotic catheter platform based on previous work (Rafii-Tari et al., in: MICCAI2013, pp 369–377. https://doi.org/10.1007/978-3-642-40763-5_46, 2013) by proposing a method to address anatomical variability among aortic arches. It incorporates anatomical information in the process of catheter trajectories optimization, hence can adapt to the scale and orientation differences among patient-specific anatomies.

**Methods:**

Statistical modeling is implemented to encode the catheter motions of both proximal and distal sites based on cannulation data obtained from a single phantom by an expert operator. Non-rigid registration is applied to obtain a warping function to map catheter tip trajectories into other anatomically similar but shape/scale/orientation different models. The remapped trajectories were used to generate robot trajectories to conduct a collaborative cannulation task under flow simulations. Cross-validations were performed to test the performance of the non-rigid registration. Success rates of the cannulation task executed by the robotic platform were measured. The quality of the catheterization was also assessed using performance metrics for manual and robotic approaches. Furthermore, the contact forces between the instruments and the phantoms were measured and compared for both approaches.

**Results:**

The success rate for semiautomatic cannulation is 98.1% under dry simulation and 94.4% under continuous flow simulation. The proposed robotic approach achieved smoother catheter paths than manual approach. The mean contact forces have been reduced by 33.3% with the robotic approach, and 70.6% less STDEV forces were observed with the robot.

**Conclusions:**

This work provides insights into catheter task planning and an improved design of hands-on ergonomic catheter navigation robots.

## Introduction

Endovascular intervention has become mainstay treatment for many vascular pathologies. Endovascular manipulation of catheters and guidewires by the clinician under fluoroscopy is necessary to reach target areas and deliver treatment. In recent years, there has been a growing interest in robot-assisted catheter navigation systems. Compared to manual catheterization, these platforms have potential advantages such as added stability and precision of movement, increased comfort for the operator, and reduced radiation from ionizing sources [1]. Recent advances in imaging, machine learning and robotic technologies may enhance robot-assisted catheterization. Learned motion patterns and tool movement profiles from multiple operators and demonstrations can be applied to semiautonomous robotic catheterization within different anatomical geometries. This can potentially reduce the cognitive workload of the operator while minimizing access path-related complications such as perforation, embolization, and dissection caused by excessive interactions between surgical instruments and the vasculature, especially in diseased and weakened vessels [2].

**Fig. 1:**
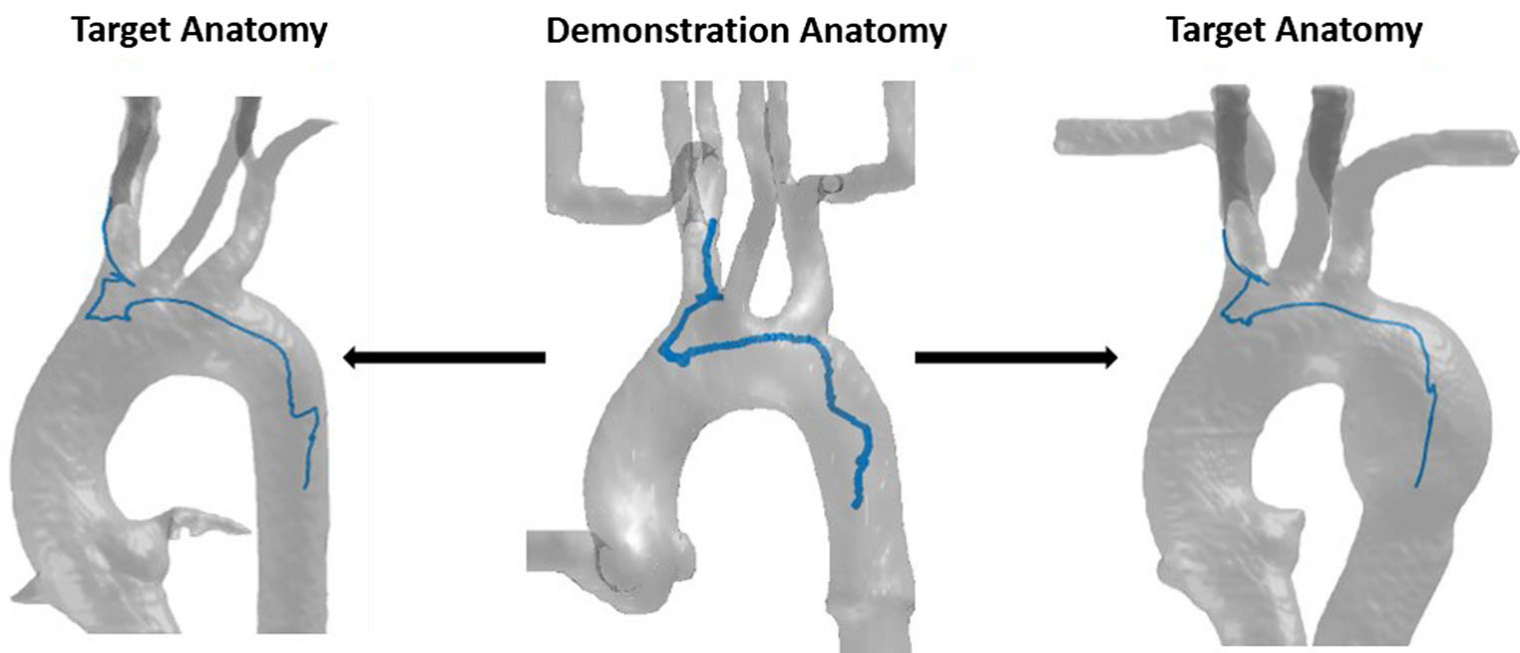
Examples of catheter tip motion (blue line) transformation from a demonstration anatomy (center) to target anatomies (left and right), the shape of the tip trajectories varies in orientation and scale after transformation

One of the most commonly used commercially available steerable platforms for endovascular intervention is the Magellan System (Hansen Medical, Mountain View, CA, USA), though many master/slave platforms have also been developed for standard catheters in the research domain [3]. Instrument manipulation is achieved through multi-DoF haptic interfaces or joysticks which alter the natural patterns of catheter manipulation, thus failing to utilize the operators experience obtained from conventional catheterization. There has been a growing interest in developing ergonomic master interfaces that can potentially utilize the experience-related skills of the endovascular interventionalist [4].

Recent research has explored the application of the “learning from demonstration” (LfD) framework, commonly used in robotics, toward automating some aspects of minimally invasive surgeries. These studies include complete automation of time-consuming and repetitive tasks [5], as well as collaborative surgery in which the control is shared back and forth between the operator and the robot [6]. Recent studies have looked into generalizing learned demonstrations to previously unseen initial conditions [7], as well as an adaptive trajectory planning to deal with dynamic changes in the environment [8]. In the field of endovascular intervention, these learning-based techniques have been used for automation of a catheterization task based on motion trajectories from expert demonstrations. These studies have demonstrated that by using a robotic driver, improvements over manual catheterization are possible [9]. Expert surgeons’ skill models were also used to train novice operators through providing haptic feedback in a customized training platform [10]. Preoperative images for surgical navigation also offer the possibility for robotic path planning based on anatomical information. Commercial robotic systems such as the Sensei X system (Hansen Medical, Mountain View, CA, USA) integrated 3D electroanatomic mapping (EAM) technology for improved navigation of the robotic catheter [11]. Other research [12] applied skeletonization techniques (as in CT angiography) to extract blood vessel centerlines, achieving efficient path planning for endovascular surgical tools. More recently, a cooperative robotic catheterization platform was developed for adapting learned trajectories to different vascular anatomies using shared control navigation [13]. However, directly integrating anatomical landmarks to aid semiautonomous robotic catheterization within different anatomical settings have not been explored as yet.

**Fig. 2:**
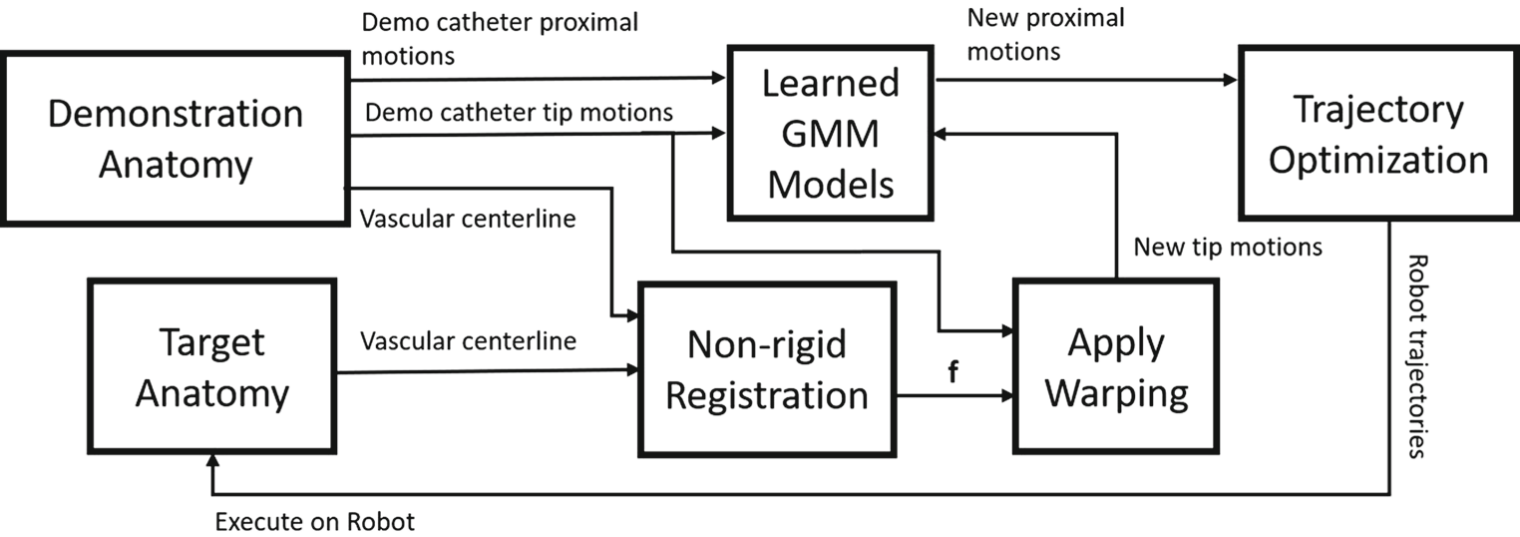
A schematic diagram of the proposed robotic trajectory optimization method by incorporating anatomical information. “Demo” is the short form for “demonstration”

In previous work [9], a LfD framework was developed to partially automate endovascular procedures through encoding and replicating operators’ hand motion patterns. This paper improves this semiautomatic robotic catheterization framework by addressing the subject-specific variability among type I aortic arches, through incorporating the anatomical information obtained from preoperative image data. In the proposed approach, catheter tip positions at the distal end and axial/rotational motions exerted by the operator at the proximal end were obtained from demonstrations performed on vascular models. These are jointly used to train statistical models that encode the essential motion patterns of the operator and the catheter motions. Together with the model’s anatomical information, a trajectory generator is proposed to generate patient-specific trajectories that can potentially tolerate catheterization task scale and orientation differences. From this, a robotic catheter control sequence can be determined for different vascular models by integrating anatomical information through non-rigid registration techniques. Figure 1 demonstrates our approach for mapping catheter tip motions to different anatomies. The approach is verified by testing the generated robotic trajectories into different vascular models with flow simulation, achieving a high success rate for cannulation tasks under continuous flow simulation. The quality of the catheterization is further assessed by comparing the proposed robotic approach against manual techniques. The robotic approach achieved smoother catheter paths, and the catheter also exerted less contact forces on the phantom, which potentially reduce the risk of complications such as perforation and dissection of diseased blood vessel. The proposed platform provides insights into endovascular task planning based on preoperative image data, and designing of a hands-on catheter navigation system that utilizes the natural skills of the operator.

## Materials and methods

An overview of the proposed methods for adapting robotic trajectories to new anatomical models is shown in Fig. 2. The details are explained in this section including the methodologies for catheter motion modeling, the transformation of the catheter tip motion, and trajectory optimization. The validation method for each module is introduced as well.

### Catheterization motion modeling

Our method for catheterization motion modeling is based on the previous work of the authors in [9]. Gaussian Mixture Models (GMM) were used to train models of catheter proximal motions and catheter tip motions jointly from demonstrations. The objectives are: (1) capture the underlying motion patterns of the catheter for a catheterization task in a specific type of aortic arch (type I); (2) encode the correlation between catheter proximal motions and tip motions; and (3) produce smoothed robotic trajectories that are executed on the robotic catheterization platform [9].

**Fig. 3:**
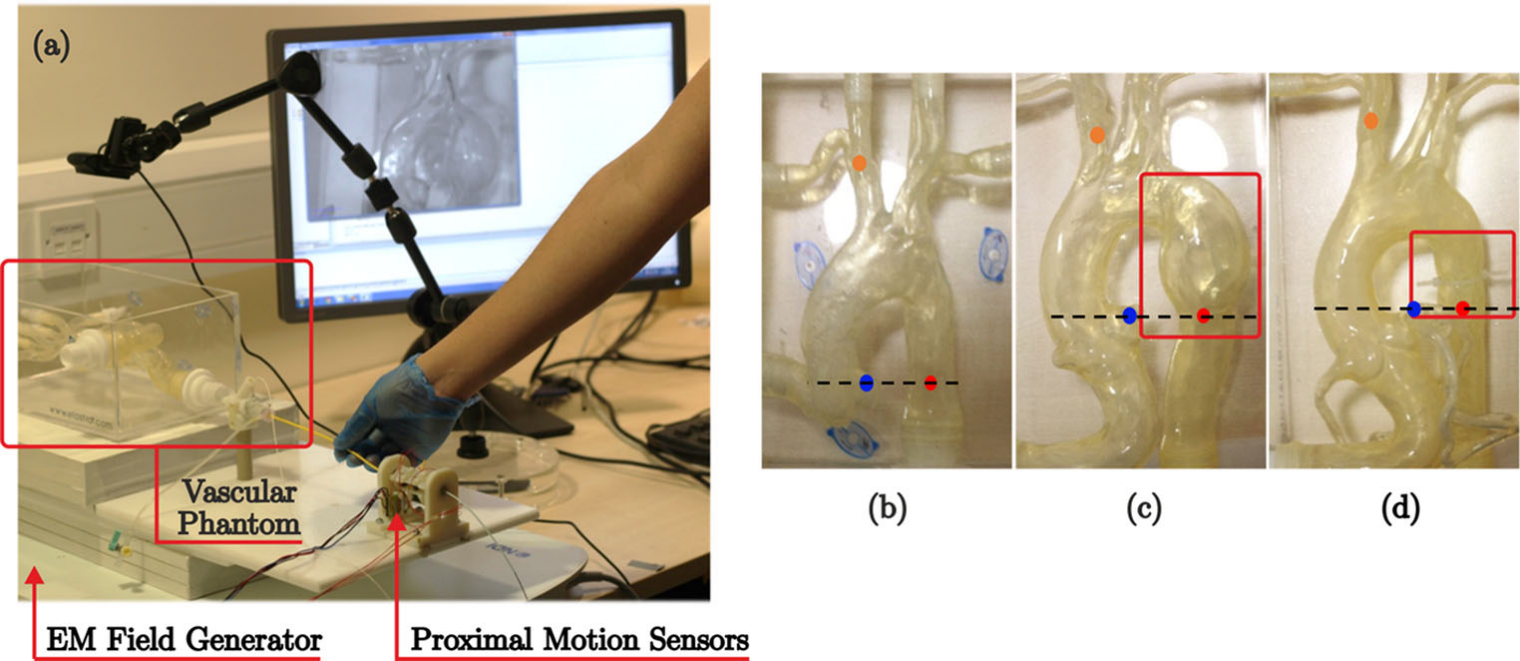
**a** The experimental setup of data collection, **b** vascular phantom with a healthy arch, **c** vascular phantom with aneurysm, **d** vascular phantom with recreated stenosis. Red and orange points represent the starting and ending positions of the procedure, respectively, whereas blue points are the positions of LCA

**Task demonstration** Catheter proximal and tip motion data were collected during catheterization tasks performed by an expert vascular surgeon (experienced more than 300 endovascular cases). The specific task consisted of cannulating the innominate artery of a silicone-based, transparent, anthropomorphic phantom, of a standard type I aortic arch. Three type I arch models were used in this study, namely a healthy arch (Phantom A) (Fig. 3b), one with an aneurysm (Phantom B) (Fig. 3c), and one with a recreated stenosis (Phantom C) (Fig. 3d) (Elastrat Sarl, Switzerland). Six demonstrations were collected from each phantom. The demonstrations from one phantom were used to train the trajectory generator, whereas demonstrations from the other two phantoms were taken to verify the performance of the robot trajectory. The starting positions of the catheter tip were aligned with the origin of the left coronary artery (LCA), whereas the ending positions of the procedure were located at the bifurcation site between the right common carotid artery and the right subclavian artery (see Fig. 3). A 5F shaped catheter and a $0.035''$ guidewire were used in this study. A camera was mounted above the vascular phantom, and 2D projected images of the phantom were then displayed on a monitor for navigation. Catheter tip positions (*x*, *y*, *z*) were collected from a six-DoF electromagnetic (EM) position sensor (Aurora, NDI) which was attached to the catheter tip. Catheter proximal motion data, which consists of two DoF axial (*d*) and rotational ($\theta$) motions of the catheter, were measured from custom-designed sensors, as presented in previous works [9], through LabVIEW (National Instruments Corp., TX, USA). Recording of catheter tip positions and the proximal motions were synchronized and sampled at a rate of 33Hz. The experimental setups for data collection are shown in Fig. 3a.

**Catheter motion modeling** The data gathered from a single demonstration are $\lambda =\{t,x,y,z,d,\theta \}$, which consist of time, catheter tip position and axial/rotational motion signals. The datasets in each demonstration were manually segmented into three procedural phases: (1) traversing the descending aorta, (2) traveling through the aortic arch, and (3) cannulation of the innominate artery. Segmented datasets from each phase were temporally aligned using Dynamic Time Warping (DTW). GMM was used to generate the probabilistic representation of the dataset. A GMM of $\textit{K}$ components can be defined as:

1 $$\begin{aligned} p(\lambda )=\sum _{k=1}^{K}p(k)p(\lambda |k) \end{aligned}$$

where *p*(*k*) is the prior. The continuous observation probability distribution is $p(\lambda |k)= \mathscr {N}(\lambda | \mu _{k},{\varSigma }_{k})$ where $\mu _{k}$ and ${\varSigma }_{k}$ are the mean and covariance matrices of the Gaussian state *k*, respectively. The GMMs were then trained by the Expectation Maximization (EM) algorithm for estimating the maximum log-likelihood of the GMM parameters. The optimal number of Gaussian components (*K*) was selected based on the Bayesian information criterion [14].

### Catheter tip motion transformation

The method for calculating the catheter tip motion transformation is based on the trajectory transfer algorithm previously reported in [7]. The aim is to map the catheter tip trajectories from the demonstration anatomy into target anatomies. New tip trajectories were used to estimate new proximal motions of the catheter for the target anatomy. Firstly, vessel centerlines that represent the essential shapes of the blood vessels were extracted from 3D meshes obtained from CT scans of all three vascular phantoms. The centerline extraction was achieved using The Vascular Modeling Toolkit (vmtk) [15]. The starting and ending positions of the centerlines extracted are the same as that of the demonstration task to ensure the centerlines of all phantoms are anatomically equivalent. Non-rigid registration was performed between centerlines of the demonstration and target anatomies, and the transformation function $\mathbf f$ was then used to warp the demonstrated tip trajectories into new anatomies. We used the coherent point drift (CPD) algorithm [16] in MATLAB for non-rigid registration. The centerline $\mathbf X$ in the demonstration anatomy consists of a $M \times D$ matrix, where *D* is the dimension of the points, and the centerline $\mathbf Y$ in the target anatomy consists of an $N \times D$ matrix. The result from the registration is to compute the warping function $\mathbf f$ that maps each point in $\mathbf X$ into the corresponding target point set $\mathbf Y$, which is equivalent to solving an optimization problem:

2 $$\begin{aligned} \underset{\mathbf{f}}{\mathbf{minimize}}\left\{ -\sum _{n=1}^{N}\log \sum _{m=1}^{M}\hbox {e}^{-\frac{1}{2}\left\| \frac{ \mathbf{x}_{n}-\mathbf{y}_{m} }{\sigma }\right\| ^{2}}\right\} +\hbox {Regularizer}\left( \mathbf{f} \right) \end{aligned}$$

where $\sigma$ is the standard deviation of each GMM (for CPD algorithm) component that is generated from both matrices, and the regularizer is a function that allows the transformation to be smooth. The transformation matrix $\mathbf f$ was then applied to warp the demonstrated trajectories.

In order to validate the accuracy of the transferred catheter tip trajectories after the registration, a cross-validation was performed. The distance values calculated by DTW were used as a measure of similarity between the simulated tip motion trajectories and the demonstrated trajectories in the same vascular model. Firstly, the distance values were calculated between each demonstrated trajectory in the same phantom, and the largest distance value was set as the limit to assess the transferred trajectories. Then, each transferred trajectory was compared to all demonstrated trajectories, and the transferred trajectory was counted as accurate if the average distance value was smaller than the limit determined from the previous step.

### Trajectory optimization and robot trajectory generation

The learned GMMs were used to estimate the axial/rotational motions from the simulated catheter tip motions after the non-rigid registration. The simulated tip positions $\xi _{t}$ were used as query points to estimate the expected corresponding spatial distribution of axial/rotational motions $\xi _{p}$ through Gaussian Mixture Regression (GMR) [14]. The conditional probability of $\xi _{p}$ with respect to $\xi _{t}$ can be defined by:

3 $$\begin{aligned} \beta _{k}=\frac{p(\xi _{t}|k)}{\sum _{i=1}^{K}p(\xi _{t}|i)} \end{aligned}$$

The obtained proximal motions were then smoothed by a further step of GMR to encode the essential features of the data into longer time steps. A new sequence of time steps is used to estimate the corresponding spatial components of the GMM. As a result, the smoothed axial/rotational motion trajectories were constructed from the training datasets in the demonstration phantom, to the other two target phantoms, for the expert operator at each segmented phase of the task.

**Fig. 4:**
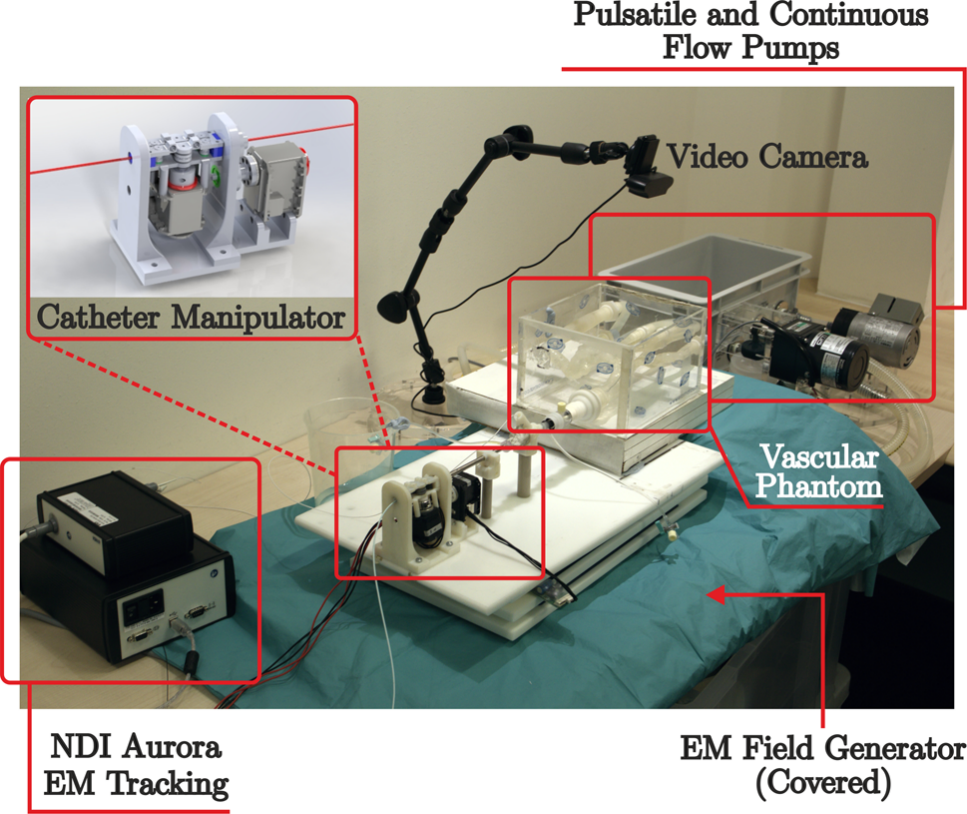
Proposed robotic platform for validation, including the CAD model of the catheter manipulator and a hybrid pump for flow simulation

The optimized proximal motion trajectories were validated by a customized robotic catheter driver to perform cannulation of the innominate artery. The robotic driver was previously reported by the authors in [9] (Fig. 4). This driver consists of two servomotors which can push/pull and rotate the catheter following the input trajectories. The catheter is driven by a pair of friction wheels that are directly coupled to one of the servomotors. The steering of the catheter is achieved by rotating the frame that holds the catheter. The robot is controlled by a PID controller. During the cannulation task, the robotic driver automates the catheter motion while an operator manipulates the guidewire for assistance. The manipulation tasks with respect to the guidewire are (1) the guidewire is stationary in procedural phase one; (2) the guidewire is inserted through the aortic arch after procedural phase one; (3) the guidewire is retracted after the procedure phase two; (4) the guidewire is inserted when catheter tip is accessed into the innominate artery during procedural phase three. The guidewire is carefully manipulated, while the catheter is stationary to avoid unwanted catheter tip movements.

Catheter motion models from demonstrations of different experience levels were used to test the proposed framework. Demonstrations were collected from novice operators ($n = 2$, male, age $=$ 24 and 27) who have no prior knowledge or experience in endovascular tasks. The novice operators learned the procedures through watching the videos of expert’s demonstrations, as well as practicing the tasks among the phantoms until repeatable skills were developed. Four demonstrations over each phantom were collected from each novice operator.

**Fig. 5:**
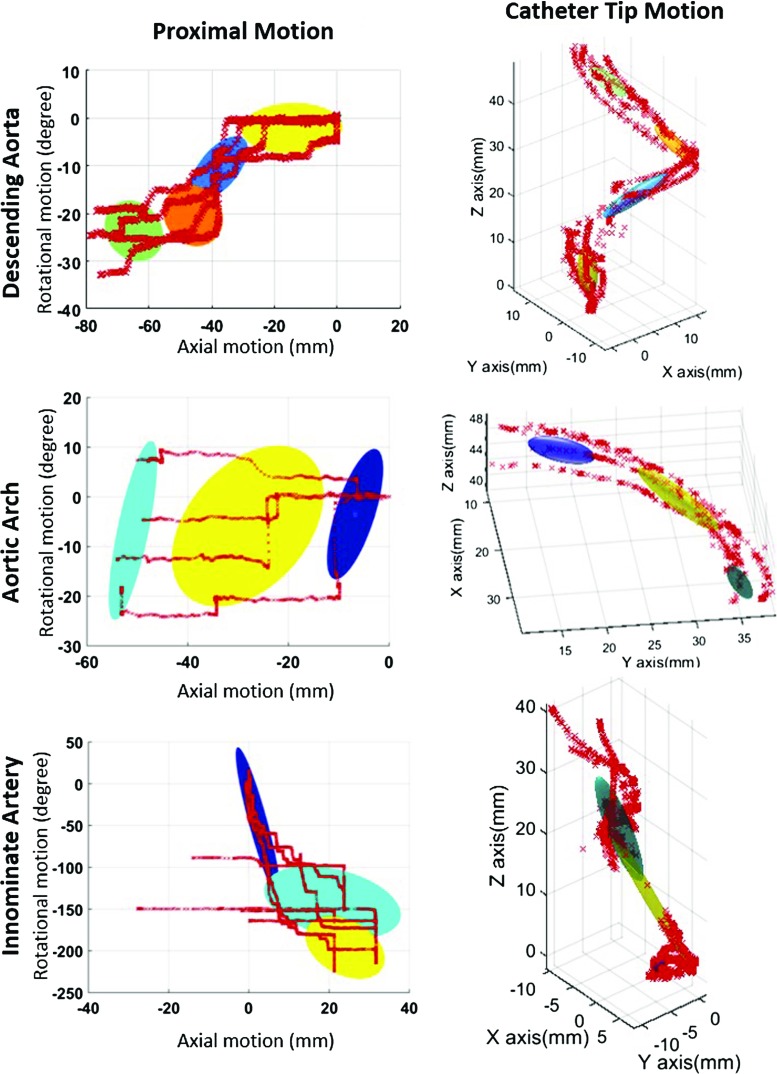
Training data from the demonstrations (red lines) and learned GMMs (colored ellipsoids) of catheter proximal motion (left) and tip motion (right)

**Table 1:** Success rate of robotic cannulation within different experience levels ,vascular models, and experimental conditions

	Dry condition (%)		Continuous flow (%)		Pulsatile flow (%)	
	CA	AR	CA	AR	CA	AR
*Expert model*						
Phantom A	100	100	100	100	100	66.7
Phantom B	100	100	100	83.3	83.3	50
Phantom C	100	100	100	100	100	50
*Novice model A*						
Phantom A	100	100	100	100	66.7	33.3
Phantom B	100	83.3	100	100	50	16.7
Phantom C	100	100	100	83.3	66.7	50
*Novice model B*						
Phantom A	100	100	100	100	50	50
Phantom B	100	83.3	83.3	66.7	33.3	16.7
Phantom C	100	100	100	83.3	66.7	50

**Fig. 6:**
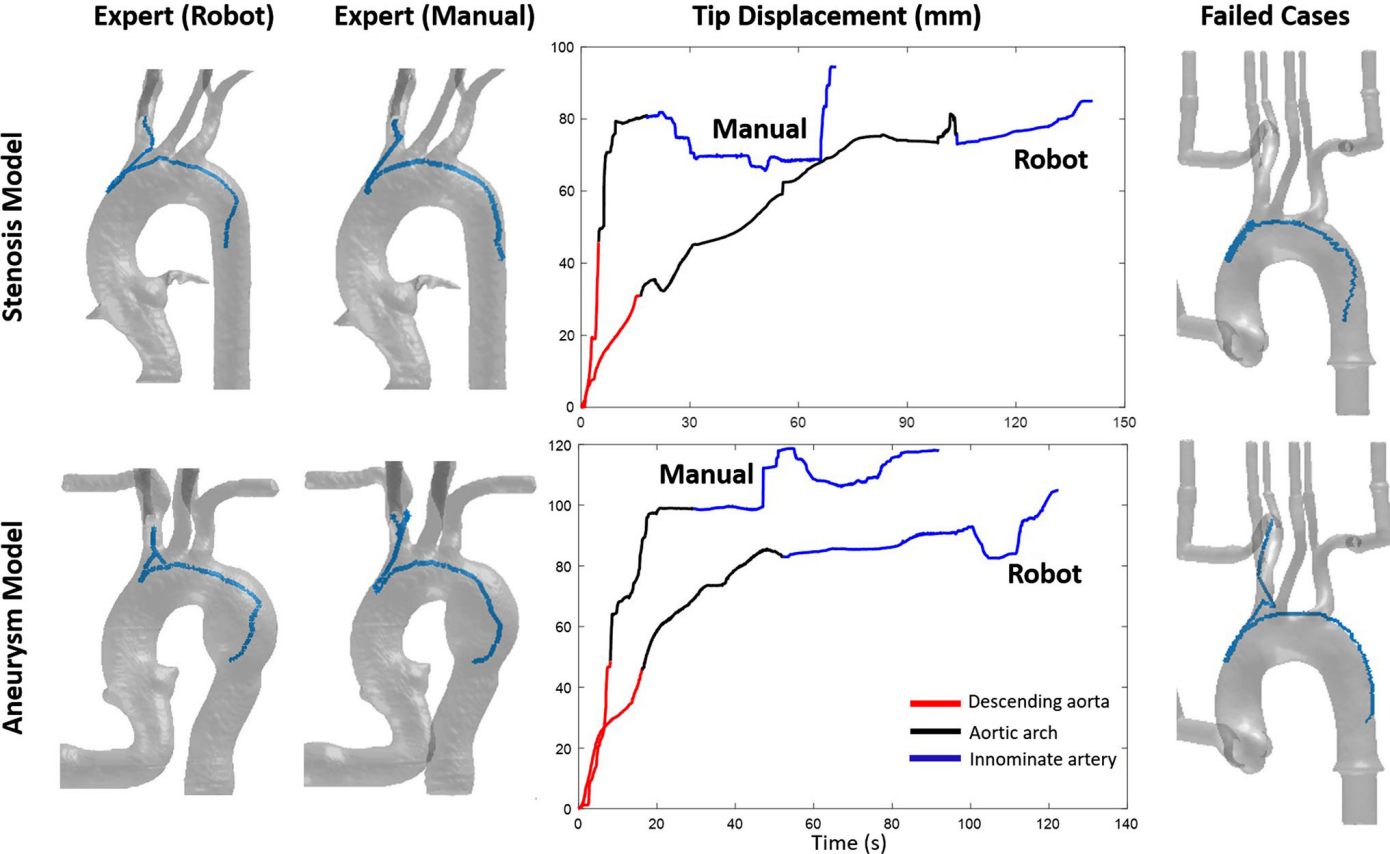
Catheter paths (left) and tip displacements (middle) obtained by the proposed robotic approach and the manual approach within different aortic arch models (Phantom B and C). Colors in the displacement graph represent segmented phases of the task. Graphs on the right show two failed cases in Phantom A under pulsatile flow simulation

“CA” is the short form for “cannulation” which represents the rate of successful cannulation to the target artery without considering the accuracy of reaching the target. An example is shown in Fig. 6 (bottom right). “AR” is the short form for “Arrival” which represents the success rate of cannulation considering the precision of the final catheter tip positions

A cross-validation was performed to find the success rate of cannulation of the innominate artery by the proposed framework. GMMs were generated from each phantom across each experience level. The demonstrated tip motions in each phantom were transferred into the other two phantoms. Robotic trajectories were estimated from each GMM and optimized for the target anatomies. The robotic driver was then used to execute the input trajectories and performs cannulation in the corresponding phantom. The robotic cannulation was carried out in each phantom under three conditions: (1) dry condition; (2) continuous flow simulation; and (3) pulsatile flow simulation. The continuous and pulsatile flow simulation conditions were achieved by using a hybrid pump (FAIN-Biomedical, Japan). The proposed robotic setup is shown in Fig. 4. A cannulation was counted as successful if the final catheter tip position was within ± 2mm of the destination position (as shown in Fig. 3). Sixty-four robotic cannulations were performed in the expert group, and 108 were performed in the novice group (six times for each phantom under each flow environment)

**Catheterization quality evaluation** The quality of catheter tip motions by the proposed robotic approach is assessed and compared against demonstrated catheter tip motions by the expert operator. In this study, the demonstration anatomy is the healthy arch (Phantom A), whereas the target anatomies are diseased phantoms (Phantom B and C). Six robotic cannulations were performed in each phantom, and tip kinematic metrics were calculated from the catheter tip trajectories. The metrics are: mean/maximum speed and acceleration, standard deviations of the speed, and total catheter path length (corresponding to the back and forth movements). All metrics over all phases were assessed using the nonparametric Wilcoxon rank-sum significance test (a value of $P<0.05$ was considered statistically significant). All data analyses were performed in MATLAB. Based on these metrics, the performance of the robotic catheterization was compared with human demonstrations in the same vascular model under dry conditions.

The contact force sensing platform developed by the authors [17] was used in this paper to measure the contact forces between the endovascular instruments and the vascular phantom. Phantom A was mounted on a plate that was fixed to a six-DoF force/torque (*F* / *T*) sensor (Mini40, ATI Industrial Automation, Inc., USA). Average root-mean-square (RMS) force modulus was calculated from the 3D forces measured by the *F* / *T* sensor. Proximal motion trajectories for Phantom A were estimated from demonstrations in Phantom B and C using the proposed trajectory generator. Those trajectories were then executed by the robot under dry condition, and the contact forces were recorded (twelve cannulations). The contact forces were then compared with that from manual catheterization, which was performed by four expert operators in the same vascular model (three cannulations per expert surgeon). The expert demonstrations were originally recorded for previous works [17]. Mean and maximum force, standard deviations of the force and force impact over time were calculated and used as quality metrics. All metrics were assessed using the nonparametric Wilcoxon rank-sum significance test.

**Table 2:** Median values for statistically significant differences ($P <0.05$) between robot-assisted learned procedures versus corresponding expert manual demonstrating data within different anatomies

Expert	Aneurysm model		Stenosis model	
	Manual	Robot	Manual	Robot
Mean speed (mm/s)	6.75	2.78	4.16	2.17
Max speed (mm/s)	356.3	124.7	255.0	177.5
STDEV speed (mm/s)	20.7	4.56	23.4	7.01
Mean acceleration (mm/s$^2$)	226.1	104.8	139.2	77.4
Max acceleration (mm/s$^2$)	$1.15\times 10^{3}$	279.6	801.8	527.3
Path length (mm)	360.5	281.2	–	–

**Fig. 7:**
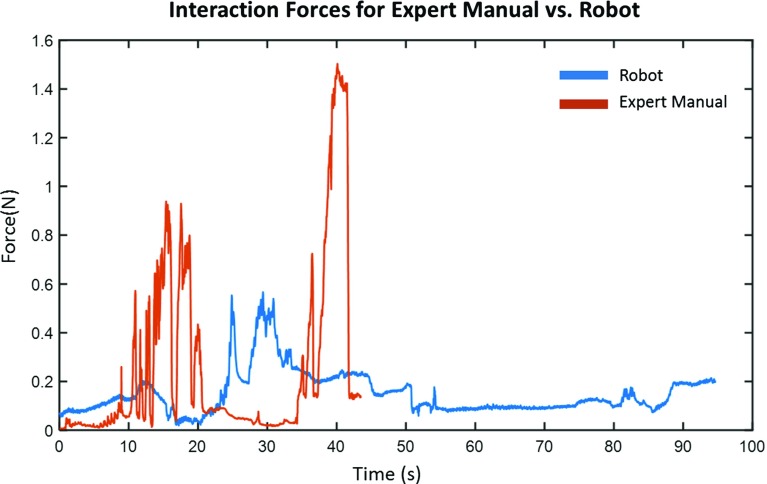
A comparison of contact forces exerted by the expert (orange color) operator and the robot (blue color) for cannulation of the innominate artery in Phantom A

## Experimental results and discussion

### Catheter motion modeling

Figure 5 shows the GMMs of both axial/rotational motion trajectories and catheter tip motion trajectories in procedural phase one (traveling through descending aorta). The colored ellipsoids represent the GMM components, which are matched between the proximal and tip motions.

### Catheter motion transformation

For the validation of the catheter motion transformation, 36 simulated tip trajectories were generated from the expert group (12 from each phantom) and 48 simulated tip trajectories were generated from the novice group (8 from each phantom). 86.1% (31/36) of the trajectories in the expert group were classified as accurate. 91.6% (44/48) trajectories from the novice dataset were classified as accurate. Results show that the majority of the demonstrated trajectories can be mapped to different anatomical settings.

### Experiments with the robotic platform

Table 1 shows the success rates of the catheterization task by the proposed robotic platform under three experimental conditions. The high success rates under dry and continuous flow conditions suggest that the proposed framework is able to adapt to anatomical variability across type I aortic arch models. This method can also be used to learn skills from operators with different experience levels. For the failed cases under continuous flow, the catheter reached beyond the target due to reduced friction caused by water. Examples of failed cases are shown in Fig. 6 (bottom right). However, under the pulsatile flow condition, many cannulation attempts failed because of the changes in the shape of the vascular phantom. An example is shown in Fig. 6 (top right); the catheter tip was stuck in the aortic arch. Future work to address dynamic movements of the phantom is briefly reported in conclusion.

Table 2 shows the result of the nonparametric test, with median values for statistically significant differences ($P <0.05$) between the manual approach and the proposed robotic approach. Compared to manual catheterization, the robot performed the catheterization at a lower speed and acceleration in the two target phantoms. In both cases, the standard deviations of the speed are significantly lower, which suggest more continuous and controlled catheter motions. The robotic approaches in Phantom B can achieve a shorter path length compared to the manual approach. These results suggest reduced back and forth movements of the catheter tip and also a reduced number of contact between the catheter and the vessel wall. Potential clinical advantages include fewer chances of tissue perforation and vessel dissection, especially in Phantom B where weakened vessel walls are presented.

Figure 6 depicts examples of the catheter paths (left) and tip displacements (middle) of the robotic and manual approaches across different phantoms. Robotic trajectories were generated from the demonstrations in Phantom A and were executed in Phantoms B and C. The robotic approach achieved smoother tip displacements than that of the manual approach. Steeper displacements in the first procedural phase and overall shorter duration of the procedure are observed in the human demonstrations.

Figure 7 shows the differences between manual and robotic approaches in terms of forces measured over time. The majority of the time, contact forces are lower with the proposed robotic platform. There is also less perturbation of forces over time. Reduced contact forces could contribute to lower risk of vessel perforation and dissection. The metrics in Table 3 provide more insight into the forces that were applied to the vascular model. Compared to manual approaches, both mean and maximum forces are significantly lower during robot-assisted catheterization. The standard deviations of the forces are significantly lower which suggest more steady and repeatable catheter motions. However, the force impact over time is higher with robotic manipulation since the procedures lasted longer.

**Table 3:** Median values for statistically significant differences ($P <0.05$) between contact forces exerted on the vasculature from robot-assisted procedures versus expert manual procedure in Phantom A

	Manual	Robotic
Mean force (N)	0.225	0.150
Maximum force (N)	1.29	0.555
STDEV force (N)	0.309	0.0907
Force impact area (N s)	8.09	16.0

## Conclusion and future work

This paper proposes an improved robotic platform for semiautonomous endovascular catheterization, using non-rigid registration to find a warping function between anatomical landmarks that can map demonstrated catheter tip trajectories into different anatomical settings. Underlying motion patterns from catheter proximal motions and tip motions were extracted and encoded by statistical modeling. Transferred tip trajectories and the learned models were used as a trajectory generator to optimize trajectories for subject-specific anatomies. Experiments show high success rates of a cannulation task by using the proposed trajectory generator and the robotic catheter driver on different aortic arch models. The quality of the robotic catheterization was assessed by comparing performance metrics derived from catheter motions to that of the manual approach. Smoother, more continuous and shorter path lengths were observed from the results, which indicate safer and more controlled catheter motions. Moreover, the proposed robotic approach is compared to the manual techniques by measuring contact forces exerted on the vasculature by the catheter. The robot achieved less mean and maximum forces than the manual approach over time and significantly smoother force patterns. The proposed methods show robust performance over three characteristic type I aortic arch models. Future improvements of the robotic platform include applying the proposed methods into more types of arch models as well as different vasculature, and incorporating dynamic shape instantiation into the proposed trajectory generator to achieve real-time trajectory optimization and adapt dynamic movements of the aorta. Moreover, integration of physiological motion simulation in the validation setups could improve the realism. The learning of catheter tip motion and proximal motion also provides insights into modeling control policies of standard catheters. The methods proposed in this paper can be further applied to other endovascular instruments and different endovascular procedures.
